# Environmental determinants of intraspecific variation in five functional traits of *Pinus yunnanensis* Franch

**DOI:** 10.3389/fpls.2024.1501584

**Published:** 2024-12-11

**Authors:** Xinrui Song, Ying Liu, Dongli Yu, Shuting Li, Wuchao Gao, Hong Zeng, Dongyu Cao, Shixing Zhou, Xinglei Cui

**Affiliations:** ^1^ National Forestry and Grassland Administration Engineering Research Centre for Southwest Forest and Grassland Fire Ecological Prevention, College of Forestry, Sichuan Agricultural University, Chengdu, China; ^2^ National Forestry and Grassland Administration Key Laboratory of Forest Resources Conservation and Ecological Safety on the Upper Reaches of the Yangtze River and Forestry Ecological Engineering in the Upper Reaches of the Yangtze River Key Laboratory of Sichuan Province, Chengdu, China

**Keywords:** *Pinus yunnanensis*, intraspecific variation, functional traits, phenotypic plasticity, adaptive traits

## Abstract

*Pinus yunnanensis* Franch. is a native species in southwestern China, characterized by high polymorphism. However, the environmental drivers of intraspecific variation in its functional traits remain poorly understood. In this study, we examined the relationships between five functional traits (bark thickness, tree height, leaf dry matter content, leaf length, and specific leaf area) and habitat conditions across 20 populations, representing three varieties: var. *yunnanensis* (the original variety), var. *pygmaea*, and var. *tenuifolia*. Our experiments aimed to determine whether the functional traits varied among the three varieties under different environmental conditions. As specific leaf area and leaf dry matter content showed no significant correlations with any environmental factors, we focused our analysis on the remaining three traits. Using random forest models, we assessed the significance of each environmental factor and found the following: Temperature seasonality was a key determinant of tree height; soil particle size (clay and sand) had the strongest influence on bark thickness; and for leaf length, precipitation during the driest quarter was the most important factor. These findings offer insights into the variation in functional traits of *P. yunnanensis* and enhance our understanding of its adaptation to diverse environments.

## 1 Introduction

Plant functional traits are any measurable attributes that directly or indirectly affect plant adaptability (Violle et al., 2007; Albert et al., 2010, 2012). Intraspecific variation in traits reflects the outcomes of evolutionary processes that individuals have responded to abiotic and biotic environmental constraints (Valladares et al., 2007). The polymorphism of traits within a species can enable individuals to adapt to diverse environmental conditions (Matías and Jump, 2014; Seidel and Menzel, 2016). For instance, *Pittosporopsis kerrii* (Icacinaceae), one of the most abundant tree species in the Xishuangbanna tropical seasonal rainforest in southwestern China, exhibits increased leaf thickness and decreased specific leaf area with increased elevation (Xu et al., 2020). *Nothofagus pumilio* (Nothofagaceae), a tree species widely distributed in southern Chile, shows a decreasing trend in leaf mass per area with increasing temperature, and a decreasing trend in wood density with increasing precipitation (Fajardo and Piper, 2011). Investigating the intraspecific variation in functional traits can provide insights into the evolutionary mechanisms of these traits and facilitate understanding of how a given plant species adapt to different environments (Turcotte and Levine, 2016; Silva et al., 2019).

**Figure 1:**
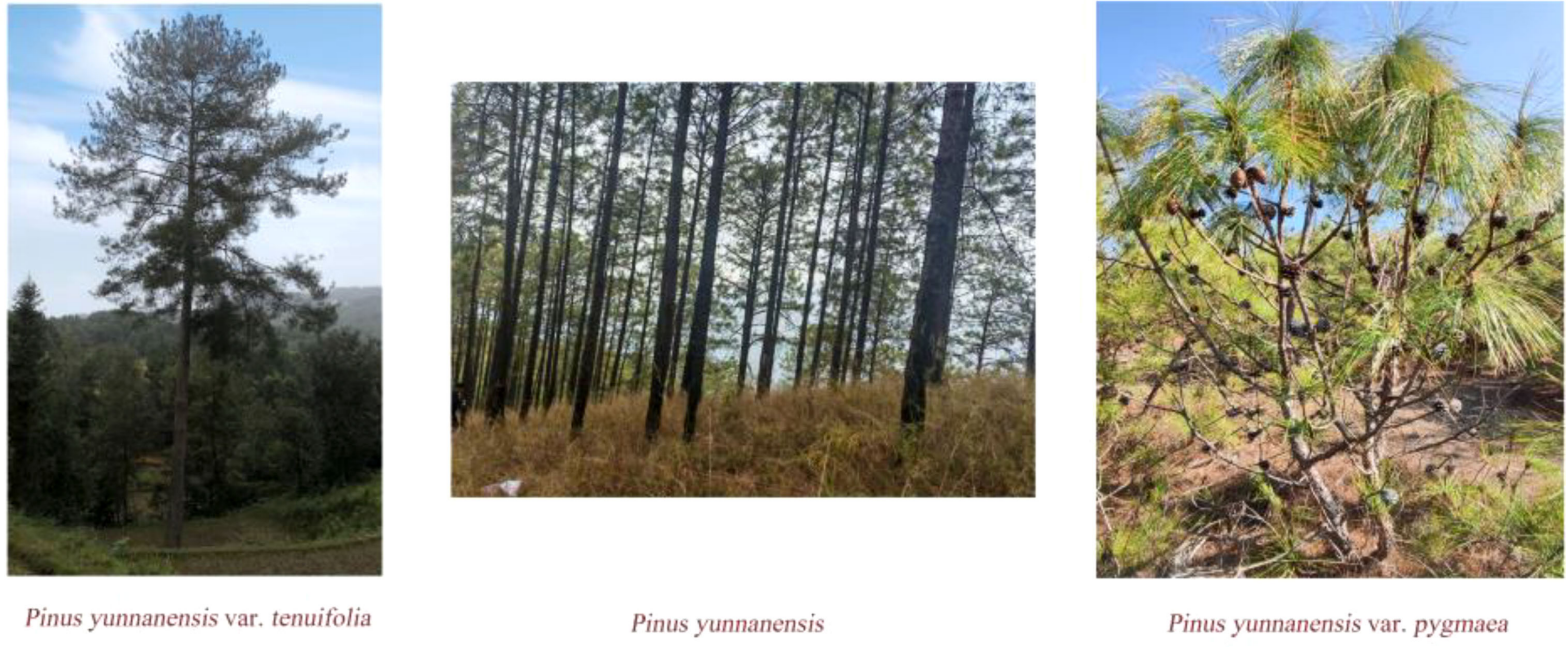
Differences in the phenotypic appearance of the three varieties of *P. yunnanensis*.

*Pinus yunnanensis*, an endemic species of southwestern China, grows in diverse geological settings and exhibits considerable intraspecific phenotypic variation (Sun et al., 2020a). *Pinus yunnanensis* is typically propagated by seeds and has winged seeds, belonging to the category of cross-pollination, with wind as the pollination medium. Due to its wide distribution and complex, diverse survival environments, it has led to the gradual independent evolution of different ecotypes in adaptation to various environments (Su et al., 2015; Xu et al., 2015, 2016a; Sun et al., 2020b). The significant differences in traits among these varieties are believed to be caused by anthropogenic factors and geographic conditions. Within the species, it exhibits significant morphological and genetic variation. Due to its wide distribution and a wide range of habitats, *P. yunnanensis* exhibits considerable intraspecific variation in many traits, resulting in three varieties: var. *yunnanensis* (the original variety), var. *pygmaea*, and var. *tenuifolia* (Pausas et al., 2021). Var. *yunnanensis* and var. *tenuifolia* are trees that generally grow above 15 m, while var. *pygmaea* is a multi-stemmed shrub that only grows around 1-3 m. In the wildfire-prone Yunnan-Guizhou Plateau region, the three variants gradually also developed different adaptation strategies, such as the arboreal variants of var. *tenuifolia* and var. *yunnanensis* which are better adapted to surface fires, and the shrubby variant of var. *pygmaea*, which is adapted to crown fires (Su et al., 2019) (**Figure 1**). The leaves of var. *yunnanensis* are typically between 10 and 30 centimeters in length, whereas var. *pygmaea* has shorter leaves, ranging from 7 to 13 centimeters; var. *tenuifolia* possesses the longest leaves, reaching up to 36 centimeters (Wang et al., 2023).

Intraspecific variation is a result of the combined effects of environmental factors, such as temperature, precipitation and soil characteristics, rather than being driven by a single factor (Piao et al., 2014; Seddon et al., 2016; Jung et al., 2017). Although the intraspecific variation in functional traits of *Pinus yunnanensis* has been widely investigated, most previous studies have been limited to specific environmental factors, which could not accurately assess the mechanism of intraspecific variations in *Pinus yunnanensis* (Han et al., 2015; Liu et al., 2023). Consequently, it is imperative to explore the impacts of various factors on the intraspecific variation observed in *Pinus yunnanensis*.

Environmental factors play an essential role in plant life and adaption (Anderson et al., 2020; Xiang et al., 2022; Aqeel et al., 2023; Chen et al., 2024). In this study, In this study, we investigated how habitat conditions(precipitation and temperature data, influence environmental variables such as nitrogen content, organic carbon content, sandy soil content, soil clay content, and coarse debris content (0.5-15.3 cm in size) in the soil) affect intraspecific variation in five functional traits (leaf length, specific leaf area, bark thickness, height, and leaf dry matter content) of *Pinus yunnanensis*. Since *Pinus yunnanensis* has evolved different variants in response to different fire behaviors in fire-prone habitats, the relevant plant height, leaf traits, and bark thickness are the traits that can better demonstrate the traits related to fire adaptation, and better help us to understand the fire adaptive responses of plants under the disturbance of fire factors. Aiming to understand how these habitat conditions collectively have shaped these traits.

## 2 Methods

### 2.1 Study sites and sample collection

**Figure 2:**
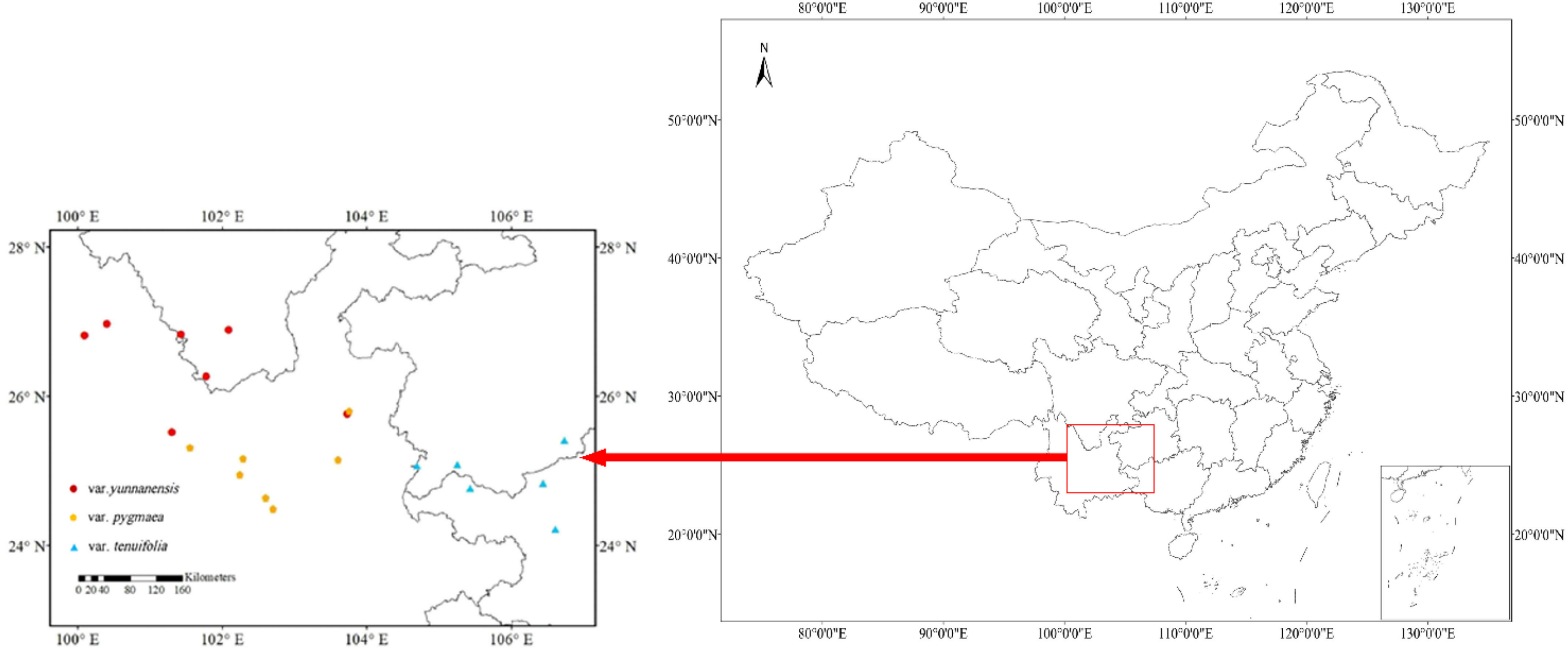
Distribution of *P. yunnanensis* populations sampled around Yunnan-Guizhou Plateau in this study shown on a map of China.

*Pinus yunnanensis* is one of the dominant species in the Central Yunnan Plateau as well as in the Western Yun-Gui Plateau. In this study, we selected 20 P*. yunnanensis* populations (7 populations for var. *yunnanensis*, 7 populations for var. *pygmaea*, and 6 populations for var. *tenuifolia*) ranging from 377 to 2555 m (**Table 1**; **Figure 2**) Mature natural forests were selected for field survey and recording during sampling, and when selecting samples, we chose mature individuals with good growth within the sample plots to ensure the accuracy and scientific validity of the study. The sampling and trait measurement methods were derived from previously published studies of congeneric species (Urbaniak et al., 2011; Xu et al., 2016a). Six healthy, mature individuals were selected from each population for sampling. Sampled trees were located at least 10 meters apart within each population. Two-year-old needles were randomly sampled from three to five vigorous branches on each sampled individual and immediately placed in labeled bags.

**Table 1:** Sampling sites in this study.

Three varieties	Name of sampling site	Longitude (°)	Latitude (°)	Altitude (m)
**var*. yunnanensis***	Miyi Datian Huimin Taian Guchengqu Yaoan Zhanyi	102.0895 101.7839 101.4331 100.0996 100.4112 101.3075 103.7331	26.8933 26.2714 26.8322 26.8181 26.9757 25.5206 25.7633	1295 1671 1399 2555 2116 2318 2031
**var*. pygmaea***	Zhanyi Luliang Jinning Liujie Lubiao Qinfeng Mouding	103.7603 103.6050 102.6043 102.7115 102.2479 102.2943 101.5580	25.8047 25.1535 24.6385 24.4934 24.9466 25.1648 25.3147	2017 1968 1895 2066 1914 1905 1767
**var*. tenuifolia***	Wusha Xinqiao Longlin Lingyun Leye Luodian	104.6936 105.2502 105.4317 106.6118 106.4370 106.7306	25.0730 25.0931 24.7689 24.2247 24.8386 25.4180	1213 1151 503 504 1230 377

### 2.2 Functional traits measurement

Tree height: Six mature individuals were selected to measure tree height at each sampling site. The measurements were carried out by using a combination of a laser rangefinder, a leveling instrument, and a leveling rod. Place the laser rangefinder at a certain distance from the tree. First, use a level and a leveling rod to determine a horizontal line and ensure that the laser rangefinder is positioned on the horizontal line; and don’t easily move the rangefinder. Aim the laser beam emitted by the rangefinder at the tree’s root position to obtain a distance parameter L_1_ and record the angle between the beam and the horizontal line, denoted as $a_{1}$ ; the distance parameter from the tree tip position to the laser rangefinder is L_2_, with the beam and the horizontal line making an angle $a_{2}$ ; and record the vertical distance from the rangefinder to the tree trunk, which is L_0_. By inputting these parameters into the following mathematical function calculation formula, Tree height (m) can be calculated as (Cornelissen et al., 2003):


$$\text{Three height }=\;L_{0}\;\times \;\tan a_{2}\;+{\text{ L}}_{1}\;\times \;\sin a_{1}$$

Bark thickness: The bark thickness was obtained by blades and saws to cut and remove, followed by using a vernier caliper for bark thickness measurement. The bark thickness of var. *yunnanensis* and var. *tenuifolia* were selected at a height of around 1.3 m above the ground. As for var. *pygmea*, due to its smaller stature, measurements were taken near ground level.

Leaf dry matter content: Fresh leaf and stem samples are washed and immersed in distilled water to ensure full saturation for 24 hours. After saturation, excess surface water was gently removed using absorbent paper, and the samples were weighed to determine their saturated mass (m_1_). The samples were then placed in an electronic drying oven at 65°C until a constant mass was achieved and reweighed (m_2_). Subsequently, leaf dry matter content (g/g) was calculated as (Polley et al., 2022):


$$LDMC=\frac{m_{2}}{m_{1}}$$

Leaf length and specific leaf area: Number the leaves in groups of six leaves each. Flatten them and place them on the scanner bed (HP Laser MFP 136w), scanning each leaf at a resolution of 300 DPI. Save the scanned images in JPEG format. Use ImageJ 1.53a (NIH, Bethesda, MD, USA) software to measure the length, width, and leaf area of each leaf in the scanned images (Katabuchi, 2015), with units in centimeters (cm) and square centimeters (cm²) respectively. The specific leaf area is the ratio of leaf area to leaf dry matter content, expressed in units of cm²/g.

### 2.3 Environmental data collection

Nineteen bioclimatic variables and latitude data were obtained from the World Climate Database (WorldClim: https://www.worldclim.org/data/index.html). We used the high spatial resolution (30 arc-s, ~1 km at the Equator) from it (Hijmans et al., 2005) The 30 arc-second resolution means that the data cover the globe in a grid of about 1 kilometer, with each grid point providing an average value of a climate variable. Soil data, including nitrogen content, organic carbon content, sandy soil content, soil clay content, and soil coarse debris content of 0.5-15.0 cm of soil at 1 km spatial resolution (Hengl et al., 2017), were obtained from SoilGrids (https://soilgrids.org/) (Chen et al., 2022). Finally, we gathered data for 24 environmental variables.

### 2.4 Data analysis

The variation of functional traits across *P. yunnanensis* intraspecific differences was analyzed by the One-way ANOVA method, and we set the significance level equal to 0.05. We used the Pearson correlation coefficient to analyze correlations between functional traits and environmental factors. The relative importance of different environmental factors for functional traits was evaluated by a random forest model in *randomForest* package (4.7-1.1). Random forest model has the highest prediction accuracy, and the random forest model of environmental factor impact traits developed accordingly has better accuracy and prediction ability than the stepwise regression model. The relative importance of each factor to functional traits is further analyzed by using the random forest regression model (GFBI consortium et al., 2019; Mantas et al., 2019; Wang et al., 2022) (**Figure 5**). All statistical analysis and plotting were performed using R 4.1.1 (R Foundation for Statistical Computing, Vienna, Austria) and Origin 2021 (Origin Lab Corporation, Northampton, MA, USA). Creating distribution maps was done using ArcGIS 10.2 (Esri, Redlands, CA, USA).

## 3 Results

### 3.1 Intraspecific variation in five functional traits within *P. yunnanensis*

**Figure 3:**
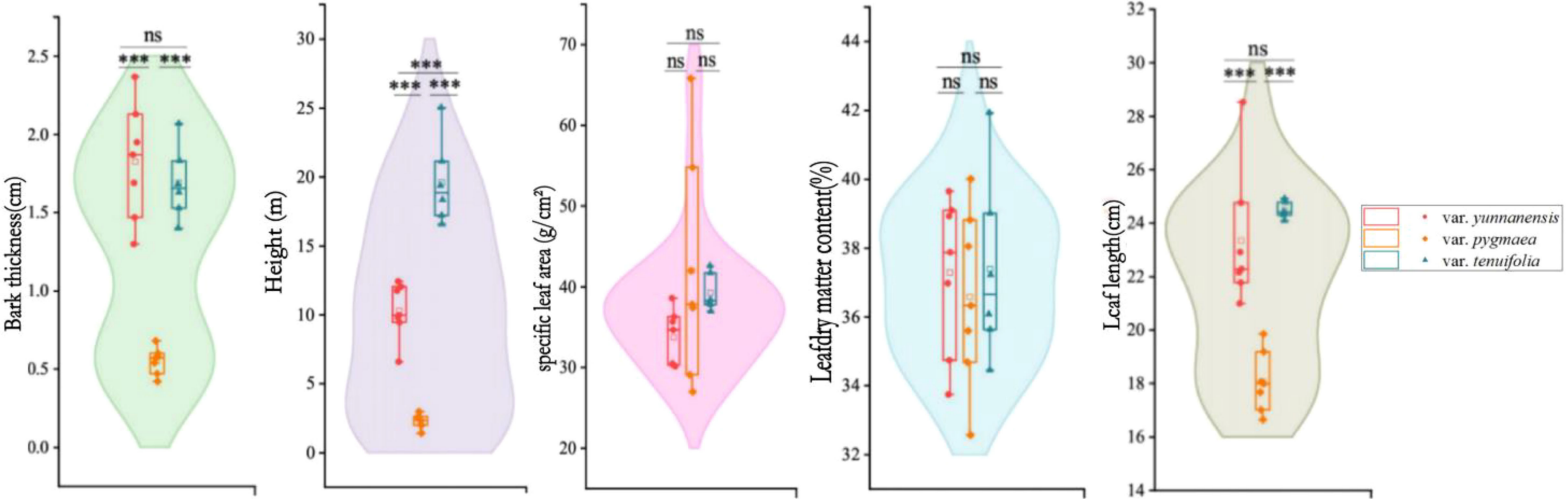
Variations in functional traits of *P. yunnanensis*. The value in this figure is expressed as mean ± SD (the same below), and the asterisk and letter indicate the significance of the difference between the traits of the three variations (∗∗∗, *p* ≤ 0.001; ns, *p* > 0.05.).

Bark thickness of var. *yunnanensis* (1.83 ± 0.54 cm) and var. *tenuifolia* (1.69 ± 0.38 cm) is significantly higher than that of var. *pygmea* (0.55 ± 0.13 cm) (*p* ≤ 0.05). Tree height of var. *tenuifolia* is 19.61 ± 3.11 m, followed by var. *yunnanensis* (1.83 ± 0.54 cm), and tree height of var. *pygmea* is only 2.28 ± 0.52 m. Leaf length of var. *pygmea* (18.06 ± 1.13 cm) is significantly shorter than that of var. *yunnanensis* (23.35 ± 2.57 cm) and var. *tenuifolia* (23.48 ± 0.30 cm) (*p* ≤ 0.001), while no significant differences are observed between var. *yunnanensis* (23.35 ± 2.57 cm) and var. *tenuifolia* (23.48 ± 0.30 cm) (*p* ≤ 0.05). Both specific leaf area and leaf dry matter content show no significant differences among the three varieties (**Figure 3**).

### 3.2 Relationships among these five studied functional traits

**Figure 4:**
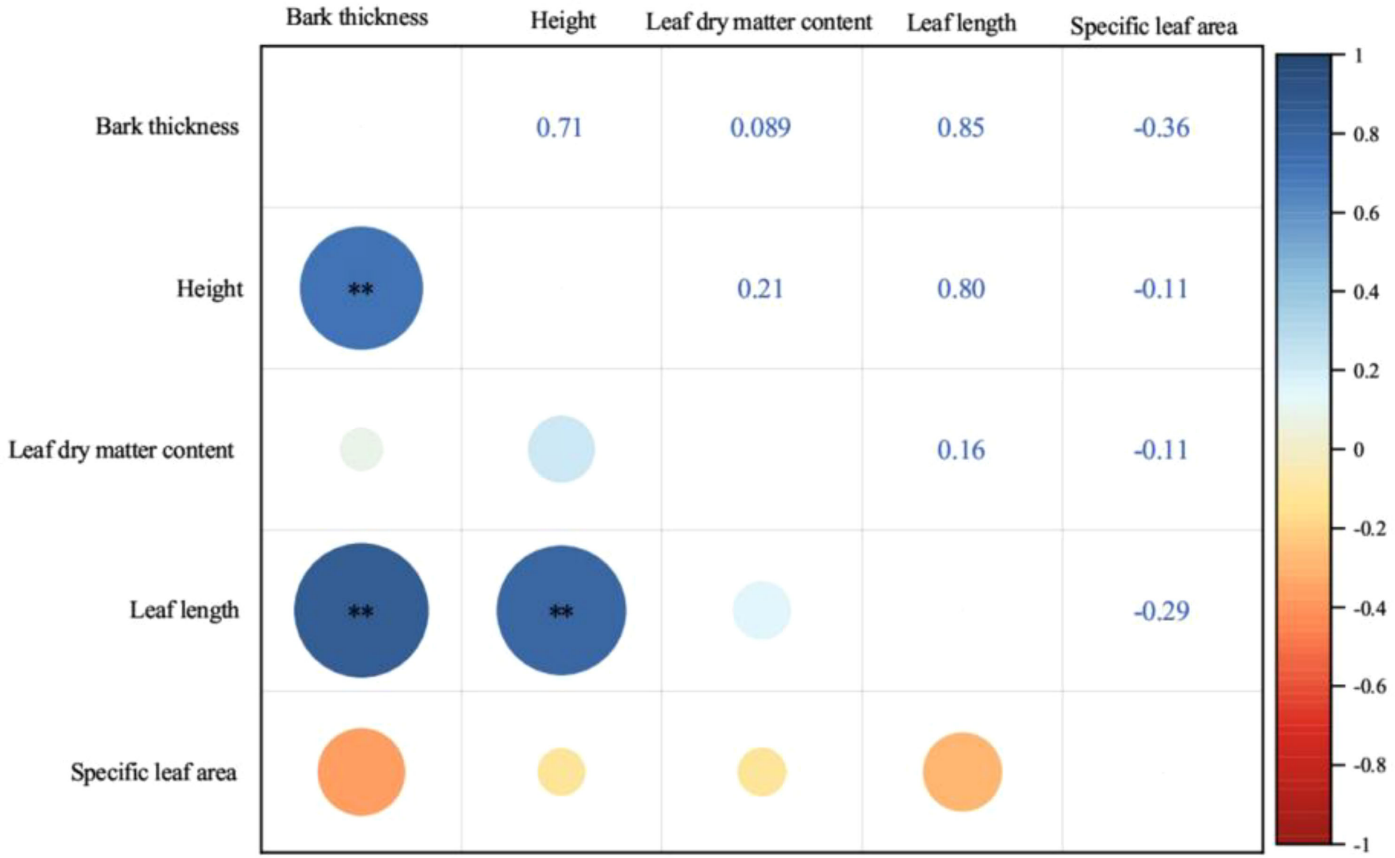
Pearson’s correlation between functional traits of *P. yunnanensis*. Blue color refers to positive correlation and red color refers negative correlation. The size of the circle marks the size of the correlation coefficient. (∗∗, *p* ≤ 0.01; unmarked, *p* > 0.05.).

There was a significant positive correlation among bark thickness, tree height and leaf length (*p* ≤ 0.01). Specific leaf area and leaf dry matter content showed no significant relationships with other functional traits (*p* > 0.05, **Figure 4**).

**Figure 5:**
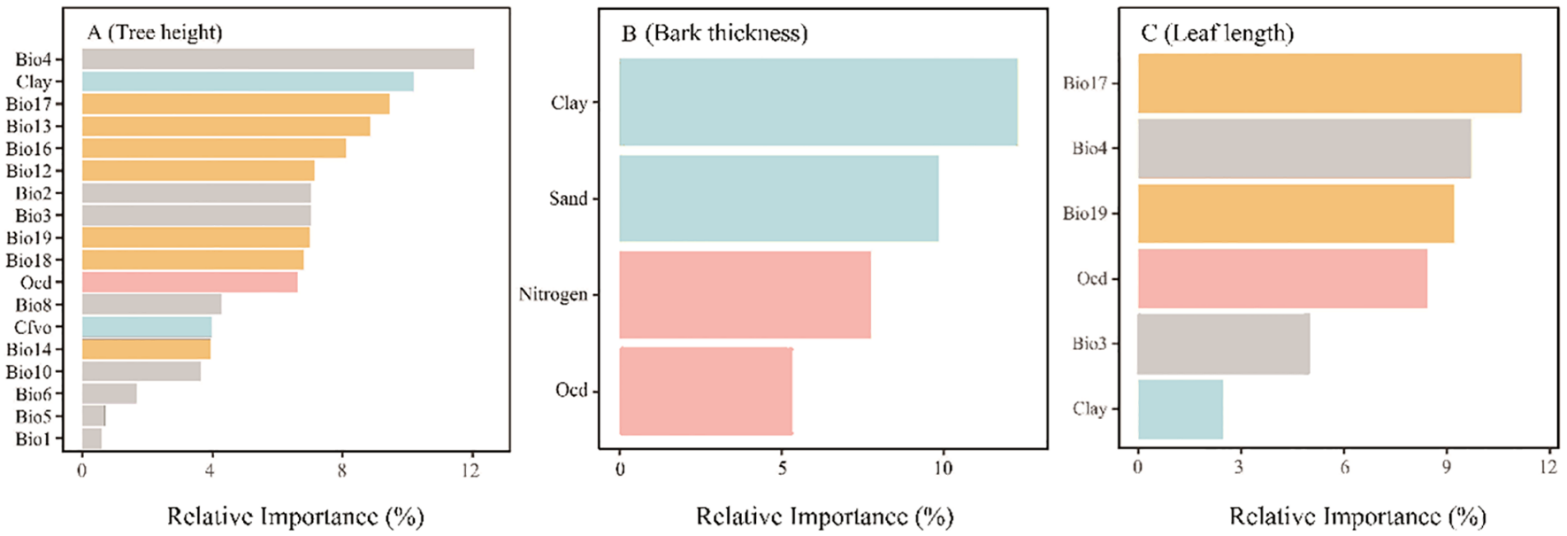
Relative Importance of different environmental factors to the Tree height **(A)**, Bark thickness **(B)** and Leaf length **(C)** in *P. yunnanensis*. Every concrete meaning of environmental variables refer to **Table 2**.

**Table 2:** Information about 24 environment variables.

Environmental variables	Description	Unit
Bio1	Annual Mean Temperature	°C
Bio2	Mean Diurnal Range	°C
Bio3	Isothermality	
Bio4	Temperature Seasonality	
Bio5	Max Temperature of Warmest Month	°C
Bio6	Min Temperature of Coldest Month	°C
Bio7	Temperature Annual Range	°C
Bio8	Mean Temperature of Wettest Quarter	°C
Bio9	Mean Temperature of Driest Quarter	°C
Bio10	Mean Temperature of Warmest Quarter	°C
Bio11	Mean Temperature of Coldest Quarter	°C
Bio12	Annual Precipitation	mm
Bio13	Precipitation of Wettest Month	mm
Bio14	Precipitation of Driest Month	mm
Bio15	Precipitation Seasonality	mm
Bio16	Precipitation of Wettest Quarter	mm
Bio17	Precipitation of Driest Quarter	mm
Bio18	Precipitation of Warmest Quarter	mm
Bio19	Precipitation of Coldest Quarter	mm
Cfvo	Vol. fraction of coarse fragments (> 2 mm)	%
Clay	Proportion of clay particles (< 0.002 mm)	%
Nitrogen	Total nitrogen (N)	g/kg
Ocd	Organic carbon density	Kg/m^3^
Sand	Proportion of sand particles (> 0.05 mm)	%

19 bioclimatic variables were extracted from WorldClim (https://www.worldclim.org/data/index.html), these environment variables use the 30 arc-s version of the study.

### 3.3 Environmental determinants of intraspecific variation in functional traits

Specific leaf area and leaf dry matter content were not related to any environmental factor.

Tree height had a significant relationship with most environmental factors, including significant positive correlations with annual mean temperature, temperature seasonality, max temperature of warmest month, min temperature of coldest month, mean temperature of wettest quarter, mean temperature of warmest quarter, annual precipitation, precipitation of wettest month, precipitation of driest month, precipitation of wettest quarter, precipitation of driest quarter, precipitation of warmest quarter, precipitation of coldest quarter, and organic carbon density, while significant negative correlations with mean diurnal range, isothermality, Vol. fraction of coarse fragments and proportion of clay particles.

Bark thickness had a significant positive relationship (*p* ≤ 0.05) with total nitrogen, organic carbon density, and proportion of sand particles, while a negative relationship with proportion of clay particles. We also found that bark thickness had no significant relationship with all climatic factors.

Leaf length had a significant positive correlation with temperature seasonality, precipitation of driest quarter, precipitation of coldest quarter and organic carbon density (*p* ≤ 0.05). However, it displayed a significant negative correlation with isothermality and proportion of clay particles (*p* ≤ 0.05) (**Table 3**).

**Table 3:** Simple linear relationship between functional traits and different factors.

	Tree height (m)		Bark thickness (cm)		Leaf length (cm)		Leaf dry matter content (g/g)		Specific leaf area (cm²/g)	
	Slope	R^2^	Slope	R^2^	Slope	R^2^	Slope	R^2^	Slope	R^2^
Bio1	1.791*	0.240	0.047	-0.028	-0.002	0.337	0.138	-0.039	-0.198	-0.053
Bio2	-4.545***	0.608	-0.158	0.050	0.144	-1.107	-0.174	-0.046	-0.802	-0.041
Bio3	-1.175***	0.733	-0.044	0.088	-0.331*	0.262	-0.087	-0.015	-0.116	-0.050
Bio4	0.110***	0.778	0.004	0.106	0.033**	0.319	0.010	0.006	0.013	-0.048
Bio5	1.829**	0.386	0.061	0.010	0.454	0.083	0.187	-0.012	-0.154	-0.053
Bio6	1.867**	0.331	0.058	-0.007	0.357	0.016	0.083	-0.048	-0.008	-0.056
Bio7	2.710	0.037	0.136	-0.025	1.615	0.111	1.230	0.125	-1.543	-0.035
Bio8	1.937**	0.429	0.068	0.023	0.473	0.091	0.134	-0.033	-0.107	-0.055
Bio9	1.162	0.023	0.027	-0.050	0.131	-0.050	0.114	-0.048	-0.636	-0.039
Bio10	1.945***	0.463	0.065	0.020	0.467	0.096	0.166	-0.020	-0.066	-0.055
Bio11	1.057	0.014	0.015	-0.054	0.058	-0.054	0.088	-0.051	-0.522	-0.044
Bio12	0.026***	0.484	0.001	0.012	0.006	0.104	0.000	-0.054	0.006	-0.033
Bio13	0.131**	0.384	0.004	-0.014	0.027	0.040	-0.011	-0.028	0.031	-0.038
Bio14	1.070*	0.215	0.007	-0.054	0.218	0.002	0.125	-0.020	0.397	-0.030
Bio15	-0.257	0.026	-0.021	0.013	-0.165	0.115	-0.104	0.071	0.015	-0.055
Bio16	0.040**	0.347	0.001	-0.023	0.008	0.020	-0.003	-0.036	0.011	-0.035
Bio17	0.373***	0.434	0.011	0.003	0.108*	0.154	0.030	-0.026	0.075	-0.042
Bio18	0.038**	0.359	0.001	0.017	0.008	0.032	-0.003	-0.035	0.004	-0.052
Bio19	0.346**	0.414	0.014	0.045	0.122*	0.242	0.034	-0.014	0.039	-0.051
Cfvo	-1.266**	0.322	-0.062	0.065	-0.416	0.151	0.091	-0.037	0.222	-0.048
Clay	-0.842*	0.231	-0.063*	0.156	-0.404**	0.280	-0.170	0.056	0.278	-0.034
Nitrogen	0.973	-0.053	0.881*	0.175	3.275	0.067	-0.131	-0.055	-1.112	-0.054
Ocd	23.156**	0.425	1.600*	0.245	9.627**	0.366	0.384	-0.054	6.285	-0.031
Sand	0.611	0.073	0.074*	0.197	0.320	0.123	0.105	-0.019	-0.375	-0.022

NA values mean there are no significant linear relationship between functional traits and factors. Every concrete meaning of environmental variables refer to **Table 2**. (*, p ≤ 0.05; **, p ≤ 0.01; ***, p ≤ 0.001; unmarked, p > 0.05).

### 3.4 Relative importance of different environmental factors to tree height, bark thickness and leaf length

Temperature seasonality had the most relative importance to tree height, explaining 12.06%, while the relative importance of annual mean temperature was the smallest, accounting for only 0.58%.

The relative importance of soil particle size (clay and sand) was higher in bark thickness, contributed 12.28% and 9.83%, respectively. The relative importance of total nitrogen to bark thickness is second (7.75%), and the relative importance of organic carbon density is the smallest (5.32%).

In terms of needle leaf length, precipitation of driest quarter had the highest relative importance in leaf length (11.16%), followed by temperature seasonality (9.71%), precipitation of coldest quarter (9.21%), organic carbon density (8.42%), and isothermality (4.98%). The relative importance of clay was the least (2.48%).

## 4 Discussion

We found that some functional traits of *P. yunnanensis* exhibited high variability among different populations and varieties. For instance, our research showed substantial differences in the thickness and width of *P. yunnanensis* var. *tenuifolia* needles among seven populations with distinct geographical origins, noting that the needles of this variety were smaller compared to those of *P. yunnanensis*. Similarly, Huang et al. (Huang et al., 2016) observed considerable variation in the leaf length of *P. yunnanensis* within seven different populations, with a coefficient of variation (CV) of 11.1%, which is comparable to our result of 15.1%. In addition to five functional traits, some other traits of *P. yunnanensis* also showed significant intraspecific differences, for example, the length, width, and weight of the cones (Xu et al., 2016b); Seed length and width (Cai et al., 2016); and the needle bundle also changed (Wang et al., 2004). The multiple functional traits of *P. yunnanensis* exhibit significant intraspecific variation, making it become an ideal material for studying the species’ evolution. Intraspecific variation represents the foundation of biological evolution and constitutes a crucial adaptive strategy for species to cope with environmental changes (Shen et al., 2020; Huang et al., 2022). By investigating the phenomenon of intraspecific variation in functional traits, we can gain insight into the adaptive strategies that operate within species and the diversity of genetic traits.

The study demonstrated that there were significant correlations and close coordination among different traits within the species of *P. yunnanensis*. This correlation between traits has been previously identified in other studies (Messier et al., 2010; Anderegg et al., 2018). Our findings indicate that bark thickness tends to increase in taller trees, this trait imparts mechanical strength and drought protection to the trunk (Paine et al., 2010; Brando et al., 2012). Furthermore, Leaf length is positively correlated with both tree height and bark thickness, reflecting the greater light availability in taller trees, which in turn affects leaf characteristics (Niinemets and Kull, 1995). These findings reflect the common changes in tree height, bark thickness, and needle length. Our results strongly support the existence and rationality of a system of associations between plant resource allocation and growth strategies (Reich, 2014; Zhao et al., 2016).

The results of our study indicate a significant correlation between functional traits and environmental factors. This correlation encompasses both long-term selection of genotypes by environmental factors (genetic differentiation) and individual plasticity responses to environmental factors (Lebedeva and Gerasimova, 2009; Stamp and Hadfield, 2020). Previous studies have also got similar conclusions (Wright et al., 2005; Markesteijn et al., 2010; Vasey et al., 2022). The functional traits of *P. yunnanensis* were found to be positively correlated with precipitation, temperature, temperature seasonality, organic carbon content, and organic carbon density in soil, but negatively correlated with proportion of clay particles and Vol. fraction of coarse fragments. These findings are consistent with those of other ecological inquiries. For example, the wood density of *Pinus sylvestris* was found to be positively correlated with precipitation (Laforest-Lapointe et al., 2014), while the leaf porosity of the holly was proportional to precipitation (Niinemets, 2015). The environmental factors that had the greatest influence on the intraspecific variation of functional traits of *P. yunnanensis* were elucidated in the random forest importance ranking. Our study confirm that temperature and moisture levels in the environment are the most important factors affecting its variability. Other studies have had similar results (Zhao et al., 2008; Jankowski et al., 2019; Popović et al., 2022). Specific leaf area and leaf dry matter content are weakly correlated with environmental factors. We know that specific leaf area can be calculated as leaf area/leaf dry weight, so one possible reason for the weak correlation of specific leaf area with environmental factors is that leaf area and leaf dry weight are almost directly covariant, a possibility that also applies to leaf dry matter content. In addition, the interaction of climatic and soil factors on intraspecific variation in plant traits (Ordoñez et al., 2009) may counteract each other’s effect on specific leaf area and leaf dry matter content. The convergence of these findings emphasizes the complex interactions between functional traits and environmental conditions, highlighting the critical role of phenotypic plasticity in the adaptive strategies and ecological distribution of species (Nicotra et al., 2010; Valladares et al., 2014; Henn et al., 2018). In the context of global climate change, phenotypic plasticity may be an important mechanism for organisms to adapt to new environmental conditions (Alberto et al., 2013; Donelson et al., 2018) and thus merits further investigation. Although this study focused on the effects of climatic and soil factors on differences in plant traits, the potential effects of environmental factors such as light and altitude on plant growth and acclimatization have not been considered. Light directly affects the rate of photosynthesis in plants, which in turn acts on leaf area and morphology (Tan et al., 2022), while changes in altitude may indirectly affect plant physiological traits through changes in temperature, barometric pressure, and radiation (Daunicht and Brinkjans, 1996; Rahman et al., 2020). Future studies could incorporate these factors into the analysis to gain a more comprehensive understanding of the mechanisms of plant trait adaptation.

This reveals the existence of more complex and coordinated phenotypes than those found in single-spectrum or one-trait cluster approaches. This coordinated covariation is the result of natural selection, and other combinations of traits may occur under different environmental conditions (Negret et al., 2015). Only 5 traits were selected for comparative study in this research, and other traits may also be correlated (Cai et al., 2016), so this study has certain limitations. In light of the potential for sustained alterations in environmental conditions within the distribution range of the *Pinus yunnanensis*, the coordinated covariation of functional traits may serve to enhance the species’ capacity for adaptation to environmental change (Laforest-Lapointe et al., 2014; Carvalho et al., 2020; Jiang et al., 2021). Consequently, an examination of the intraspecific variations in traits and an investigation of the relationship between trait variation and environmental factors will provide insights into the evolutionary process of the species, thereby facilitating an understanding of how the species evolved to adapt to diverse environments (Jung et al., 2010; Zuleta et al., 2022).

## 5 Conclusion

Plant functional traits are key attributes indicative of plant growth conditions, and these traits are individually or jointly adapted to the external environment and change in response to environmental changes. Incorporating intraspecific variation in plant functional traits into ecological studies can better reveal species interactions, population responses to spatial and temporal environmental gradients. In this study, our results suggest that different environmental conditions induce intraspecific variation in *P. yunnanensis*, five functional traits differed significantly among the three varieties, providing evidence for coordinated differences in traits and their covariates under external conditions. The correlations among the tree functional traits reveal that intraspecific variation in plant functional traits is also not the result of a single environmental factor, but is subject to a combination of several different environmental factors. Variation in plant functional traits across environmental gradients is driven by biotic and abiotic factors. The molecular mechanisms underlying intraspecific variation and other trait variations in *P. yunnanensis* are still unclear. To better understand the intraspecific variatis of *P. yunnanensis*, these aspects need further research.

## Data Availability

The original contributions presented in the study are included in the article/supplementary material. Further inquiries can be directed to the corresponding author.
